# Association Between Cardiovascular-Kidney-Metabolic Syndrome and Myocardial Injury After Noncardiac Surgery: A Retrospective Cohort Study

**DOI:** 10.1016/j.jacasi.2026.01.023

**Published:** 2026-03-14

**Authors:** Guosong Liao, Kai Zhang, Jiaxuan Peng, Chang Liu, Bingbing Meng, Siyi Yao, Zhao Li, Jingsheng Lou, Yanhong Liu, Jiangbei Cao, Weidong Mi, Hao Li, Qiang Fu

**Affiliations:** a Department of Anesthesiology, The First Medical Center, Chinese People's Liberation Army (PLA) General Hospital, Beijing, China; b Medical School of Chinese PLA General Hospital, Beijing, China; c Nankai University, Nankai District, Tianjin, China; d National Clinical Research Center for Geriatric Diseases, Chinese PLA General Hospital, Beijing, China

**Keywords:** cardiovascular-kidney-metabolic syndrome, myocardial injury after noncardiac surgery, perioperative complications, risk prediction

## Abstract

**Background:**

Cardiovascular-kidney-metabolic (CKM) syndrome represents a complex interplay among obesity, metabolic dysfunction, kidney disease, and cardiovascular disease. The relationship between CKM staging and myocardial injury after noncardiac surgery (MINS) has not been comprehensively studied.

**Objectives:**

This study sought to investigate the association between the CKM syndrome and MINS risk in patients undergoing noncardiac surgery.

**Methods:**

This single-center retrospective cohort study included 25,040 patients aged ≥45 years who underwent noncardiac surgery between January 2019 and December 2023. Patients were classified according to CKM stages 0 to 4. MINS was defined as postoperative troponin elevation with ischemic etiology within 30 days. Four progressive multivariable logistic regression models were constructed, and subgroup analyses were performed stratified by age, sex, and surgery type.

**Results:**

Among 25,040 patients (median time to MINS: 2.0 days [IQR: 0.8-6.6]), CKM stages 0 to 4 comprised 13.0%, 15.0%, 43.7%, 18.7%, and 9.7%, respectively. MINS occurred in 1,782 patients (7.12% [6.80-7.44]), demonstrating a J-shaped distribution: lowest in stage 1 at 141 of 3,754 (3.76% [3.19-4.41]), intermediate in stages 0 (166 of 3,246, 5.11% [4.40-5.93]) and 2 (586 of 10,943, 5.36% [4.94-5.80]), and highest in stages 3 (512 of 4,670, 10.96% [10.09-11.90]) and 4 (377 of 2,427, 15.53% [14.14-17.04]). With stage 0 as reference, stages 3 (OR: 1.95 [1.60-2.37]) and 4 (OR: 2.16 [1.75-2.66]) independently predicted increased MINS risk (both *P* < 0.001), with significant age interaction (*P* = 0.013) showing stronger associations in younger patients.

**Conclusions:**

Advanced CKM stages independently predicted an increased risk of MINS. These findings may improve perioperative risk assessment and guide preventive strategies.

In 2023, the American Heart Association introduced a framework for understanding the complex interplay among cardiovascular disease (CVD), chronic kidney disease, and metabolic disorders through the cardiovascular-kidney-metabolic (CKM) syndrome classification.^1^ This novel staging system, ranging from stage 0 (no risk factors) to 4 (established CVD), provides a comprehensive approach for risk stratification by integrating traditional siloed conditions into a unified pathophysiological framework.^1^ The development of CKM syndrome represents a paradigm shift in cardiovascular medicine, acknowledging that these conditions share common pathways, including insulin resistance, chronic inflammation, endothelial dysfunction, and neurohormonal activation.^2^

Although the CKM staging system has been proposed for cardiovascular risk stratification in the general population, its application in perioperative medicine requires special consideration. The pathophysiological mechanisms underlying CKM syndrome, including insulin resistance, chronic inflammation, endothelial dysfunction, and neurohormonal activation, may be particularly problematic in the perioperative setting.^2^ Surgical stress imposes unique physiological challenges such as sympathetic activation, inflammatory cascades, hemodynamic fluctuations, and metabolic derangements, which can unmask subclinical CVD.^3^ Patients with CKM syndrome may experience amplified responses to these stressors due to compromised cardiovascular reserves and dysregulated metabolic pathways.

Myocardial injury after noncardiac surgery (MINS) is an ideal outcome for investigating CKM-related perioperative risks. As a sensitive marker of cardiovascular stress, MINS captures subclinical myocardial injury, which may be particularly relevant for patients with CKM syndrome and compromised cardiovascular reserves.^4^^,^^5^ Defined as troponin elevation due to myocardial ischemia within 30 days of surgery, MINS occurs in 8% to 19% of noncardiac surgical patients and predicts both short-term mortality (10% at 30 days) and long-term cardiovascular events.^4^ The pathophysiological mechanisms underlying MINS, including oxygen supply and demand mismatch, plaque rupture, and microvascular dysfunction, directly overlap with the vascular pathology observed in advanced CKM stages.^5^ Given these shared pathophysiological mechanisms, CKM staging potentially correlates with perioperative MINS risk, yet comprehensive studies examining this relationship are lacking. The only available evidence comes from the correspondence of Roth et al^6^ in European populations, leaving the relationship in Asian surgical cohorts completely unexplored.

Therefore, this study aimed to comprehensively evaluate the association between CKM staging and MINS in a large cohort of patients undergoing noncardiac surgery, hypothesizing that the relationship might differ from the linear patterns observed in nonsurgical populations owing to the unique stressors of the perioperative environment.

## Methods

### Study design and population

**Figure 1:**
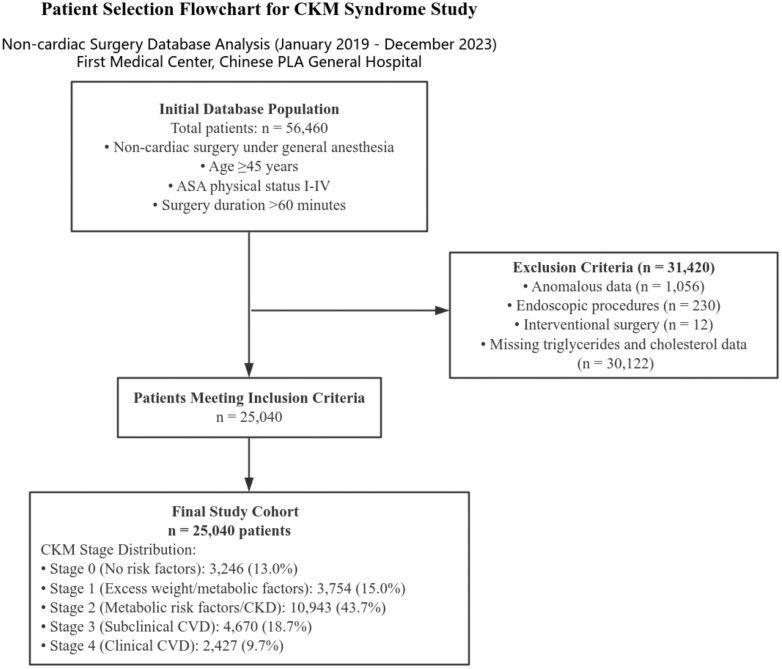
Patient Selection Flowchart Flowchart depicting patient selection from the noncardiac surgery database (January 2019-December 2023). Of 56,460 patients, 31,420 were excluded for anomalous data, endoscopic/interventional procedures, or missing laboratory values. The final cohort included 25,040 patients, with stage 2 comprising the largest proportion (43.7%). ASA = American Society of Anesthesiologists; CKD = chronic kidney disease; CKM = cardiovascular-kidney-metabolic; CVD = cardiovascular disease.

This single-center retrospective cohort study included patients aged ≥45 years undergoing noncardiac surgery under general anesthesia (>60 minutes’ duration, American Society of Anesthesiologists [ASA] classification I-IV) between January 2019 and December 2023 at the First Medical Center of PLA General Hospital. After excluding those with anomalous data, those who underwent endoscopic procedures and/or interventional surgeries, and those with missing laboratory data, 25,040 patients were analyzed (Figure 1). This study was approved by the Ethics Committee of the Chinese PLA General Hospital (S2025-271-01), with the requirement for informed consent waived due to the retrospective design.

### Data collection

Perioperative data were extracted from the hospital's electronic medical records and anesthesia information management systems. The collected variables included demographic characteristics such as age, sex, and body mass index (BMI); comorbidities, including hypertension, diabetes mellitus, hyperlipidemia, and CVD; smoking history; and alcohol consumption history. Preoperative laboratory parameters included serum creatinine, fasting blood glucose, total cholesterol, triglyceride, and troponin levels and complete blood count. In addition, surgery-related information was collected, including the ASA physical status, surgery type, duration of surgery, duration of anesthesia, urine output, blood loss, colloid and crystalloid infusion volumes, hypotensive episodes, and anesthetic medications.

### CKM stage classification

Patients were categorized into 5 CKM stages according to the American Heart Association 2023 consensus statement,^1^ Each patient was assigned to the highest applicable stage based on their preoperative clinical presentation, and all classifications were verified by 2 independent reviewers. In cases in which staging discrepancies arose between reviewers, a senior physician adjudicator made the final determination to ensure accuracy and consistency:

• Stage 0: No CKM risk factors, BMI <23 kg/m^2^, no metabolic risk factors, and no chronic kidney disease or CVD
• Stage 1: Overweight/obesity (BMI ≥23 kg/m^2^) or abdominal obesity (waist circumference ≥80/90 cm for female/male patients) and/or fasting blood glucose ≥100-124 mg/dL or HbA1c 5.7%-6.4% without metabolic risk factors
• Stage 2: Metabolic risk factors (hypertriglyceridemia, hypertension, metabolic syndrome, diabetes) or moderate to high-risk chronic kidney disease
• Stage 3: Subclinical CVD diagnosed by coronary artery calcification or elevated cardiac biomarkers (N-terminal pro–B-type natriuretic peptide ≥125 pg/mL) or subclinical heart failure
• Stage 4: Clinical CVD (coronary heart disease, heart failure, stroke, peripheral arterial disease), further divided into stage 4a (without renal failure, glomerular filtration rate ≥15 mL/min) and stage 4b (with renal failure, glomerular filtration rate <15 mL/min)

### Outcome definition

The primary outcome was MINS, defined as myocardial injury due to ischemia occurring within 30 days of noncardiac surgery. MINS was diagnosed when at least 1 postoperative high-sensitivity troponin T measurement was 20.0 to 64.9 ng/L with an absolute change of ≥5 ng/L, or when a high-sensitivity troponin T concentration ≥65 ng/L was obtained with a presumed ischemic etiology, regardless of clinical symptoms or electrocardiographic changes. Elevated troponin levels attributable to nonischemic mechanisms, including pulmonary embolism, sepsis, and renal failure, were not classified as MINS.^4^^,^^5^

The secondary outcome was postoperative acute kidney injury (AKI), which was defined as AKI occurring within 7 days of surgical intervention according to the Kidney Disease: Improving Global Outcomes (KDIGO) criteria. According to the KDIGO criteria, AKI is the presence of any of the following: increase in serum creatinine level by ≥0.3 mg/dL within 48 hours, increase in serum creatinine level to ≥1.5 times baseline within 7 days, or urine volume <0.5 mL/kg/h for ≥6 hours.^7^^,^^8^

### Statistical analysis

Normally distributed continuous variables are presented as mean ± SD, non-normally distributed continuous variables as median [IQR], and categorical variables as frequency (percentage). Between-group comparisons were performed using Student's *t*-test for normally distributed continuous variables and Mann-Whitney *U* test for non-normally distributed continuous variables. For categorical variables, the chi-square test was used when expected frequencies were ≥5, and the Fisher exact test was used when expected frequencies were <5.

To evaluate the association between CKM staging and MINS, covariates were selected a priori using a theory-driven approach based on established clinical knowledge and prior literature.^4^^,^^5^^,^^9^^,^^10^ Four progressive multivariable logistic regression models were constructed. Model 1 was unadjusted (CKM stages only). Model 2 adjusted for preoperative variables (age, sex, lymphocyte count, neutrophil count, and platelet count) that have been consistently identified as risk factors for perioperative cardiovascular complications in the American Heart Association Scientific Statement^5^ and VISION (Vascular Events in Noncardiac Surgery Patients Cohort Evaluation Study).^4^ Model 3 adjusted for surgery-related variables (operation duration, blood loss, ASA classification, hypotension events, and surgery type) known to influence myocardial oxygen supply-demand balance.^5^^,^^9^ Model 4 was fully adjusted, combining all covariates from models 2 and 3. Variables directly incorporated into CKM staging definitions (fasting glucose, triglycerides, cholesterol, serum creatinine) were excluded to prevent overadjustment. Univariate analyses were performed to characterize the distribution of covariates and their crude associations with MINS (Supplemental Table 1). To avoid overfitting, an event-per-variable ratio of approximately 10:1 was maintained.

In all regression analyses, CKM stage 0 (no CKM risk factors group) was selected as the reference group to evaluate the relative MINS risk of other stages compared with the baseline of no risk factors. This approach follows the clinical practice paradigm of assessing risk relative to a healthy baseline state and facilitates the observation of risk progression through the CKM stages.

Subgroup analyses were performed according to age (<65 or ≥65 years), sex, and type of major surgery (neuro, hepatobiliary, urological, or gastrointestinal surgery). The interaction terms between the CKM stages and subgroup variables were incorporated into the regression models to test for effect modifications.

To address potential heterogeneity within CKM stage 0, we conducted a prespecified sensitivity analysis. Patients in stage 0 were stratified by BMI into underweight (<18.5 kg/m^2^) and normal-weight (18.5–22.9 kg/m^2^) subgroups using Asian-specific cutoffs. MINS rates were compared between the subgroups using chi-square tests. Four progressive multivariable logistic regression models were constructed with normal-weight stage 0 as the reference group, consistent with the main analysis approach: model 1 was unadjusted; model 2 adjusted for preoperative variables (age, sex, lymphocyte count, neutrophil count, and platelet count); model 3 adjusted for surgery-related variables (operation duration, blood loss, ASA classification, hypotension events, and surgery type); and model 4 was fully adjusted for all covariates. Stage 1 patients were included as additional comparators to evaluate the obesity paradox.

All statistical analyses were performed using Python 3.11 with the pandas, numpy, statsmodels, and scipy packages. Statistical significance was defined as a 2-sided *P* value of <0.05.

## Results

### Baseline characteristics

Among the 25,040 patients included in the final analysis, the distribution across CKM stages was as follows: stages 0, 1, 2, 3, and 4 in 3,246 (13.0%), 3,754 (15.0%), 10,943 (43.7%), 4,670 (18.7%), and 2,427 (9.7%) patients, respectively. Table 1 presents the baseline characteristics of the patients stratified by the CKM stage.

**Table 1:** Baseline Characteristics of Patients With Different CKM Stages

	Stage 0 (n = 3,246)	Stage 1 (n = 3,754)	Stage 2 (n = 10,943)	Stage 3 (n = 4,670)	Stage 4 (n = 2,427)	*P* Value
Demographics						
Age, y	60.10 ± 9.27	59.67 ± 8.72	61.68 ± 8.79	61.64 ± 9.45	67.11 ± 8.91	<0.001
Sex						<0.001
Male	1,636 (50.4)	2,154 (57.4)	6,455 (59.0)	2,766 (59.2)	1,681 (69.3)	
Female	1,610 (49.6)	1,600 (42.6)	4,488 (41.0)	1,904 (40.8)	746 (30.7)	
BMI (kg/m^2^)	20.82 ± 1.65	25.03 ± 1.34	24.18 ± 2.32	28.35 ± 3.97	25.22 ± 3.41	<0.001
Smoking history						<0.001
Yes	768 (23.7)	857 (22.8)	3,111 (28.4)	1,264 (27.1)	927 (38.2)	
No	2,478 (76.3)	2,897 (77.2)	7,832 (71.6)	4,464 (72.9)	1,490 (61.8)	
Alcohol history						<0.001
Yes	842 (25.9)	1,034 (27.5)	3,511 (32.1)	1,474 (31.6)	931 (38.4)	
No	2,404 (74.1)	2,720 (72.5)	7,432 (68.9)	4,196 (68.4)	1,496 (61.6)	
Comorbidities						
Hypertension	0 (0.0)	0 (0.0)	5,992 (54.8)	2,226 (47.7)	1,556 (64.1)	<0.001
Diabetes	0 (0.0)	0 (0.0)	2,842 (26.0)	924 (19.8)	759 (31.3)	<0.001
Hyperlipidemia	0 (0.0)	0 (0.0)	102 (0.9)	36 (0.8)	34 (1.4)	<0.001
CVD	0 (0.0)	0 (0.0)	0 (0.0)	0 (0.0)	2,427 (100.0)	<0.001
Lab parameters						
Cr (μmol/L)	68.00 (59.8-78.30)	71.50 (61.50-81.78)	72.30 (61.90-84.10)	73.40 (61.60-87.10)	76.00 (64.70-88.85)	<0.001
FBG (mmol/L)	4.78 (4.45-5.18)	4.93 (4.60-5.37)	5.16 (4.71-5.95)	5.28 (4.76-6.14)	5.28 (4.75-6.22)	<0.001
TC (mmol/L)	4.29 (3.73-4.88)	4.31 (3.76-4.89)	4.41 (3.80-5.10)	4.33 (3.67-5.00)	3.80 (3.16-4.54)	<0.001
TG (mmol/L)	0.94 (0.75-1.17)	1.06 (0.85-1.26)	1.61 (1.14-2.10)	1.47 (1.07-2.02)	1.28 (0.94-1.79)	<0.001
Neut (×10^9^/L)	0.57 (0.51-0.65)	0.57 (0.51-0.63)	0.58 (0.52-0.64)	0.59 (0.53-0.66)	0.59 (0.53-0.66)	<0.001
Lymphocyte(×10^9^/L)	0.314 ± 0.097	0.318 ± 0.089	0.311 ± 0.088	0.293 ± 0.098	0.289 ± 0.092	<0.001
PLT (×10^9^/L)	217 (178-261)	214 (178-256)	219 (181-261)	218 (178-261)	206 (167-249)	<0.001
Surgical characteristics						
ASA Class						<0.001
I	97 (3.0)	100 (2.7)	183 (1.7)	58 (1.2)	9 (0.4)	
II	2,870 (88.4)	3416 (91.0)	9,651 (88.2)	3,862 (82.7)	1,506 (62.1)	
III	271 (8.3)	229 (6.1)	1,088 (9.9)	702 (15.0)	879 (36.2)	
IV	8 (0.2)	9 (0.2)	21 (0.2)	48 (1.0)	33 (1.4)	
Surgery duration, min	207.74 ± 115.48	204.55 ± 112.04	198.46 ± 110.04	200.39 ± 111.20	181.71 ± 92.07	<0.001
Surgery type						<0.001
Neurosurgery	765 (23.6)	974 (25.9)	2,298 (21.0)	885 (19.0)	446 (18.4)	
Hepatobiliary surgery	507 (15.6)	489 (13.0)	1,525 (13.9)	686 (14.7)	221 (9.1)	
Urology	444 (13.7)	631 (16.8)	2,414 (22.1)	1,005 (21.5)	602 (24.8)	
Vascular	27 (0.8)	40 (1.1)	267 (2.4)	109 (2.3)	232 (9.6)	
Orthopedics	156 (4.8)	270 (7.2)	828 (7.6)	569 (12.2)	171 (7.0)	
Gynecology	79 (2.4)	89 (2.4)	191 (1.7)	85 (1.8)	41 (1.7)	
Otolaryngology	118 (3.6)	115 (3.1)	314 (2.9)	88 (1.9)	35 (1.4)	
Thoracic	90 (2.8)	136 (3.6)	285 (2.6)	97 (2.1)	65 (2.7)	
Gastrointestinal surgery	849 (26.2)	801 (21.3)	2,170 (19.8)	835 (17.9)	541 (22.3)	
Thyroid and breast	123 (3.8)	167 (4.4)	497 (4.5)	238 (5.1)	56 (2.3)	
Others	88 (2.7)	42 (1.1)	154 (1.4)	73 (1.6)	17 (0.7)	
Urine output, mL	400 (100-887)	400 (100-900)	300 (100-800)	300 (100-700)	300 (100-70)	<0.001
Blood loss, mL	100 (50-200)	100 (50-200)	50 (50-200)	100 (50-200)	50 (50-150)	<0.001
Hypotension events	1,991 (61.3)	2176 (58.0)	6,270 (57.3)	2720 (58.2)	1322 (54.5)	<0.001
Outcomes						
MINS	166 (5.1)	141 (3.8)	586 (5.4)	512 (11.0)	377 (15.5)	<0.001
AKI	174 (5.4)	209 (5.6)	980 (9.0)	571 (12.2)	276 (11.4)	<0.001
ICU admission	834 (25.7)	955 (25.4)	2,429 (22.2)	1,151 (24.6)	642 (26.5)	<0.001

Values are mean ± SD (normally distributed continuous variables), median (Q1-Q3) (non-normally distributed continuous variables), or n (%) for categorical variables. Other surgery types cover ophthalmology, plastic surgery and stomatology. *P* values were calculated using analysis of variance (for means) or Kruskal-Wallis test (for medians) for continuous variables and chi-square test for categorical variables.

AKI = acute kidney injury; ASA = American Society of Anesthesiologists; BMI = body mass index; Cr = creatinine; CKM = cardiovascular-kidney-metabolic; CVD = cardiovascular disease (including coronary heart disease, heart failure, stroke, and peripheral arterial disease); FBG = fasting blood glucose; Hb = hemoglobin; ICU = intensive care unit; MINS = myocardial injury after noncardiac surgery; Neut = neutrophil; PLT = platelet; TC = total cholesterol; TG = triglycerides.

Patients with higher CKM stages were older, with the highest mean age observed in stage 4 (67.11 ± 8.91 years). The proportion of male patients increased progressively across stages, reaching 69.3% in stage 4 compared with 50.4% in stage 0. BMI demonstrated a nonlinear trend, with the highest values in stage 3 (28.35 ± 3.97 kg/m^2^). The prevalence of comorbidities, including hypertension, diabetes mellitus, and hyperlipidemia, increased with higher CKM stages as anticipated by the definition.

Laboratory parameters revealed that patients in advanced CKM stages had higher serum creatinine and fasting blood glucose levels. Regarding the ASA physical status, the proportion of patients with ASA III and IV status was notably higher in stage 4 (36.2% and 1.4%, respectively). The distribution of surgery types varied across the CKM stages, with vascular surgery being more prevalent in stage 4 (9.6% vs 0.8% in stage 0).

### Clinical outcomes across CKM stages

**Figure 2:**
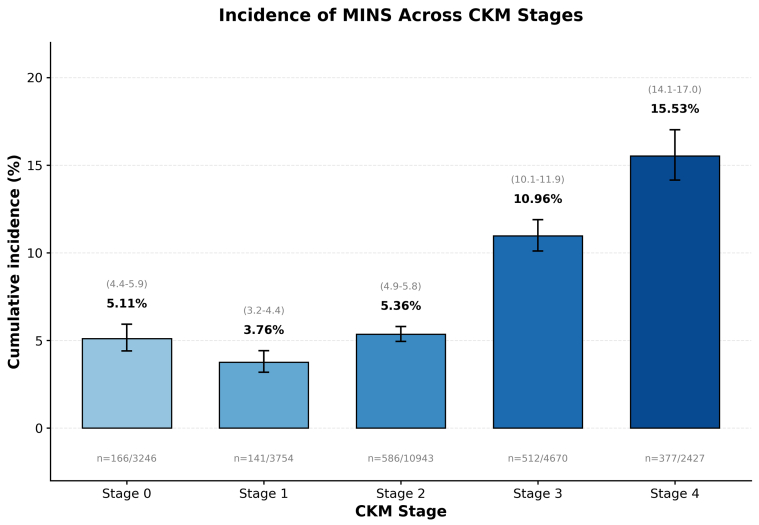
J-Shaped Distribution of MINS Incidence Across CKM Stages MINS incidence with 95% CIs across CKM stages in 25,040 patients. A J-shaped distribution was observed, with stage 1 showing the lowest rate (3.76%) and progressive increases through stages 2 to 4. The steepest rise between stages 2 and 3 identifies stage 3 as a critical risk threshold. Error bars: 95% CI (Wilson method). MINS = myocardial injury after noncardiac surgery; other abbreviation as in Figure 1.

In total, 1,782 MINS events were identified, representing an overall incidence of 7.12% (95% CI: 6.80%–7.44%). The median time from surgery to MINS occurrence was 2.0 days (IQR: 0.8–6.6), with 61.2% of events occurring within 3 days and 91.6% within 14 days postoperatively. The incidence of MINS showed a clear J-shaped distribution across the CKM stages (Figure 2, *P* < 0.001). Patients in stage 1 demonstrated the lowest incidence of MINS at 141 of 3,754 (3.76%; 95% CI: 3.19%–4.41%), which was significantly lower than stage 0 at 166 of 3,246 (5.11%; 95% CI: 4.40%–5.93%) and all subsequent stages. The incidence progressively increased through stage 2 at 586 of 10,943 (5.36%; 95% CI: 4.94%–5.80%), stage 3 at 512 of 4,670 (10.96%; 95% CI: 10.09%–11.90%), and stage 4 at 377 of 2,427 (15.53%; 95% CI: 14.14%–17.04%). The steepest risk increase occurred between stages 2 and 3, representing a relative increase of 104%.

Secondary outcomes also demonstrated significant associations with CKM staging (all *P* < 0.001). AKI occurred in 2,210 of 25,040 patients (8.83%; 95% CI: 8.48%–9.18%) and showed a similar progressive pattern across CKM stages: 174 of 3,246 (5.36%; 95% CI: 4.64%–6.19%), 209 of 3,754 (5.57%; 95% CI: 4.88%–6.35%), 980 of 10,943 (8.96%; 95% CI: 8.43%–9.50%), 571 of 4,670 (12.23%; 95% CI: 11.32%–13.20%), and 276 of 2,427 (11.37%; 95% CI: 10.17%–12.70%) for stages 0 to 4, respectively. Intensive care unit admission occurred in 6,011 of 25,040 patients (24.01%; 95% CI: 23.48%–24.54%) and varied across stages (22.2%–26.5%) without a clear progressive pattern.

### Association between CKM staging and MINS

Table 2 presents the results of the 4 logistic regression models used to examine the association between CKM staging and MINS. In the unadjusted model (model 1), compared with stage 0 (reference), stage 1 demonstrated a protective effect (OR: 0.72; 95% CI: 0.58-0.91; *P* = 0.006), stage 2 showed no significant association (OR: 1.05; 95% CI: 0.88-1.25; *P* = 0.590), and stages 3 (OR: 2.28; 95% CI: 1.91-2.74; *P* < 0.001) and 4 (OR: 3.41; 95% CI: 2.82-4.13; *P* < 0.001) were significantly associated with an increased risk for MINS.

**Table 2:** Association Between CKM Stage and MINS Risk: Results from Multivariable Logistic Regression Analysis

CKM Stage	Model 1 (Unadjusted)		Model 2 (Preoperative Adjusted)		Model 3 (Surgery Adjusted)		Model 4 (Fully Adjusted)	
	OR (95% CI)	*P* Value	OR (95% CI)	*P* Value	OR (95% CI)	*P* Value	OR (95% CI)	*P* Value
0	1.00 (reference)	—	1.00 (reference)	—	1.00 (reference)	—	1.00 (reference)	—
1	**0.72 (0.58-0.91)**	**0.006**	**0.78 (0.62-0.99)**	**0.040**	0.79 (0.63-1.01)	0.057	0.84 (0.66-1.08)	0.17
2	1.05 (0.88-1.25)	0.59	0.98 (0.82-1.17)	0.81	1.13 (0.94-1.35)	0.21	1.05 (0.87-1.26)	0.64
3	**2.28 (1.91-2.74)**	**<0.001**	**1.92 (1.59-2.31)**	**<0.001**	**2.20 (1.81-2.66)**	**<0.001**	**1.95 (1.60-2.37)**	**<0.001**
4	**3.41 (2.82-4.13)**	**<0.001**	**2.16 (1.77-2.63)**	**<0.001**	**2.85 (2.32-3.51)**	**<0.001**	**2.16 (1.75-2.66)**	**<0.001**

Model 1 was unadjusted (CKM stage only); model 2 was adjusted for preoperative variables and blood parameters (age, sex, lymphocytes, neutrophils, platelet count); model 3 was adjusted for surgery-related variables (operation duration, blood loss, ASA classification, hypotension events, surgery type); model 4 was fully adjusted for all aforementioned variables. CKM stage 0 served as the reference group. **Bold** values indicate statistical significance (*P* < 0.05).

Abbreviations as in Table 1.

After adjusting for preoperative variables (model 2), the protective effect of stage 1 persisted (OR: 0.78; 95% CI: 0.62-0.99; *P* = 0.040), stage 2 remained nonsignificant (OR: 0.98; 95% CI: 0.82-1.17; *P* = 0.810), and stages 3 (OR: 1.92; 95% CI: 1.59-2.31, *P* < 0.001) and 4 (OR: 2.16; 95% CI: 1.77-2.63; *P* < 0.001) remained significantly associated with an increased risk for MINS.

When adjusting for surgery-related variables (model 3), stage 1 showed a trend toward protection but without statistical significance (OR: 0.79; 95% CI: 0.63-1.01; *P* = 0.057), stage 2 remained nonsignificant (OR: 1.13; 95% CI: 0.94-1.35; *P* = 0.210), and stages 3 (OR: 2.20; 95% CI: 1.81-2.66; *P* < 0.001) and 4 (OR: 2.85; 95% CI: 2.32-3.51; *P* < 0.001) demonstrated strong associations with the risk for MINS.

In the fully adjusted model (model 4), the association between stage 1 and MINS was reduced and no longer statistically significant (OR: 0.84; 95% CI: 0.66-1.08; *P* = 0.170), Stage 2 also showed no significant association (OR: 1.05; 95% CI: 0.87-1.26; *P* = 0.640), whereas stages 3 (OR: 1.95; 95% CI: 1.60-2.37; *P* < 0.001) and 4 (OR: 2.16; 95% CI: 1.75-2.66; *P* < 0.001) remained independent risk factors for MINS.

### Subgroup analyses

**Figure 3:**
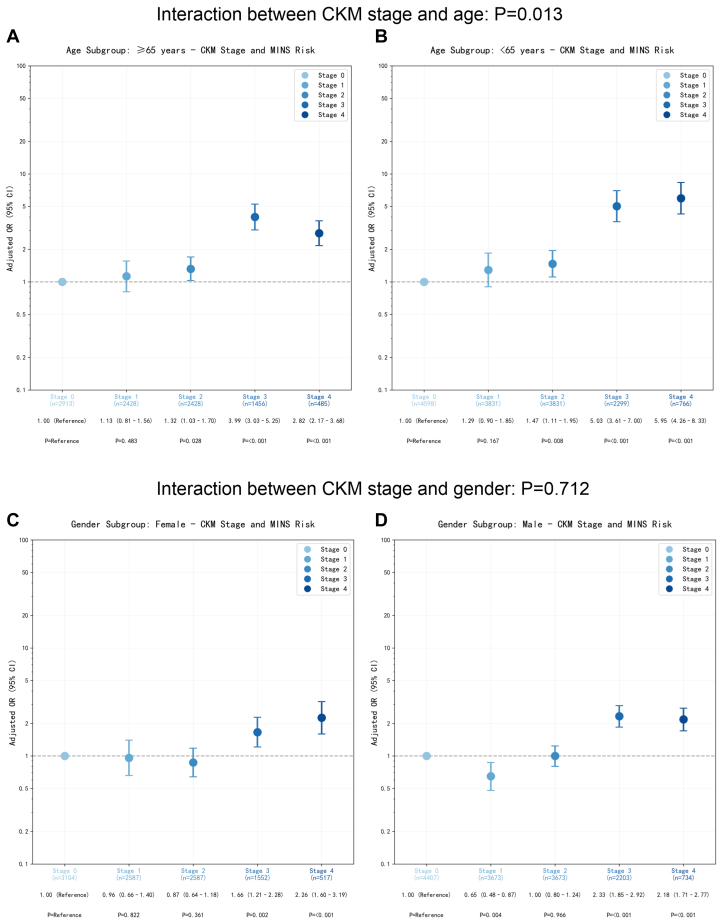

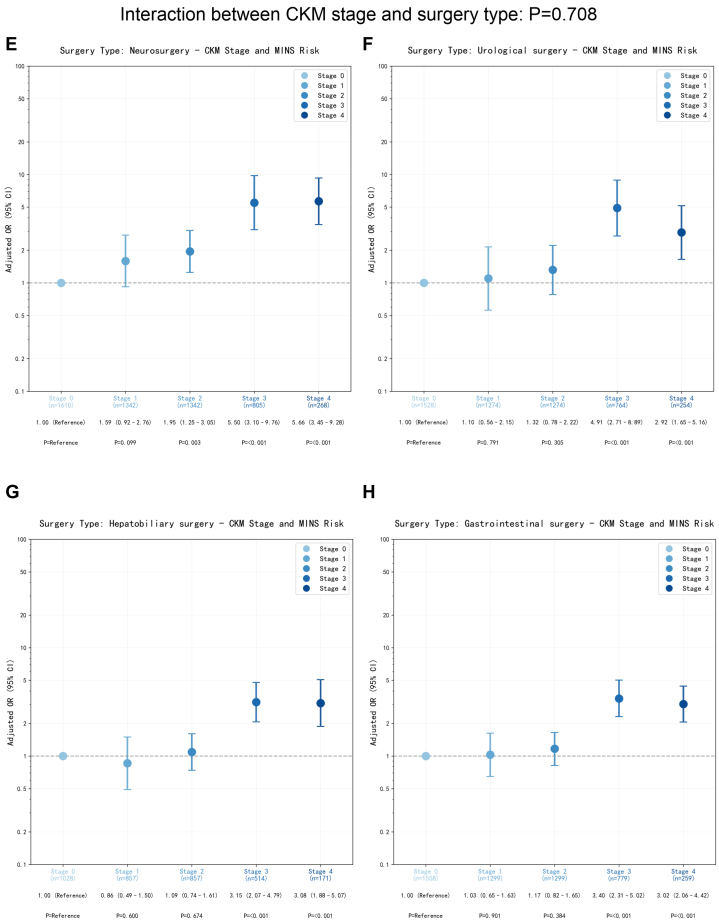
Subgroup Analyses of CKM-MINS Association Forest plots showing adjusted ORs for MINS across CKM stages, stratified by age (A, B), sex (C, D), and surgery type (E-H). Significant age interaction was observed (*P* = 0.013), with stronger associations in patients <65 years. No significant interactions were found for sex (*P* = 0.712) or surgery type (*P* = 0.708). Stage 0 served as reference (OR: 1.00). Point estimates with 95% CIs; logarithmic scale. Abbreviations as in Figures 1 and 2.

Prespecified subgroup analyses were conducted to evaluate potential effect modifications by demographic and clinical factors. Significant interaction was observed between CKM staging and age (*P* for interaction = 0.013), with stronger associations between advanced CKM stages and MINS in patients aged <65 years compared with those aged ≥65 years (Figures 3A and 3B). In patients aged <65 years, stages 3 and 4 were associated with a substantially increased risk for MINS (OR: 5.03; 95% CI: 3.61-7.00; *P* < 0.001 and OR: 5.95; 95% CI: 4.26-8.33; *P* < 0.001, respectively) compared with the reference stage 0. The associations remained significant in patients aged ≥65 years, although the effect size was reduced for stage 4 (stage 3: OR: 3.99; 95% CI: 3.03-5.25; *P* < 0.001; stage 4: OR: 2.82; 95% CI: 2.17-3.68; *P* < 0.001).

No significant interaction was observed between CKM staging and sex (*P* for interaction = 0.712) (Figures 3C and 3D). Both male and female patients showed similar patterns of progressively increasing MINS risk with advancing CKM stage, with the clearest risk elevation at stages 3 and 4. Male patients demonstrated substantial risk increases for stage 3 (OR: 2.33; 95% CI: 1.85-2.92; *P* < 0.001) and stage 4 (OR: 2.18; 95% CI: 1.71-2.77; *P* < 0.001), whereas female patients showed comparable patterns (stage 3: OR: 1.66; 95% CI: 1.21-2.28; *P* = 0.002; stage 4: OR: 2.26; 95% CI: 1.60-3.19; *P* < 0.001).

When stratified by surgery type, no significant interaction was observed between CKM staging and surgical specialty (*P* for interaction = 0.708), suggesting consistent risk associations across different procedures (Figures 3E to 3H). Neurosurgical procedures showed strong associations between CKM staging and MINS risk, particularly for stages 3 (OR: 5.50; 95% CI: 3.10-9.76; *P* < 0.001) and 4 (OR: 5.66; 95% CI: 3.45-9.28; *P* < 0.001). Comparable patterns, albeit with varying effect sizes, were observed across gastrointestinal (stage 3: OR: 3.40; 95% CI: 2.31-5.02; *P* < 0.001; Stage 4: OR: 3.02; 95% CI: 2.06-4.42; *P* < 0.001), urological (stage 3: OR: 4.91; 95% CI: 2.71-8.89; *P* < 0.001; stage 4: OR: 2.92; 95% CI: 1.65-5.16; *P* < 0.001), and hepatobiliary (stage 3: OR: 3.15; 95% CI: 2.07-4.79; *P* < 0.001; stage 4: OR: 3.08; 95% CI: 1.88-5.07; *P* < 0.001) surgeries.

### Sensitivity analysis of CKM stage 0

Among the 3,246 stage 0 patients, 341 (10.5%) were underweight and 2,905 (89.5%) had normal weight. The incidence of MINS differed significantly between subgroups: 27 of 341 (7.92%; 95% CI: 5.50%–11.27%) in underweight patients vs 139 of 2,905 (4.78%; 95% CI: 4.07%–5.62%) in normal-weight patients (*P* = 0.014). In unadjusted analysis (model 1) with normal-weight stage 0 as reference, underweight was associated with significantly increased MINS risk (OR: 1.72; 95% CI: 1.12–2.63; *P* = 0.014). The association attenuated progressively across adjusted models: model 2 (OR: 1.53; 95% CI: 0.99–2.38; *P* = 0.06), model 3 (OR: 1.52; 95% CI: 0.95–2.41; *P* = 0.08), and model 4 (OR: 1.49; 95% CI: 0.93–2.38; *P* = 0.10), likely reflecting confounding by age and surgical factors. Similarly, the apparent protective effect of stage 1 observed in model 1 (OR: 0.78; 95% CI: 0.61–0.99; *P* = 0.04) was attenuated and became nonsignificant in the fully adjusted model (OR: 0.93; 95% CI: 0.72–1.21; *P* = 0.60). Notably, underweight stage 0 patients had more than twice the MINS incidence compared with stage 1 patients (7.92% vs 3.76%, unadjusted OR: 2.20; 95% CI: 1.44–3.38; *P* < 0.001) (Supplemental Table 2).

## Discussion

This study comprehensively evaluated the association between the CKM staging system and MINS in a large Chinese surgical cohort, as summarized in the Central Illustration. Among the 25,040 patients analyzed, a distinctive J-shaped relationship emerged between the CKM stages and MINS risk, with stage 1 demonstrating a protective effect (3.76% incidence) compared with stage 0 (5.11%), whereas stages 3 and 4 showed progressively increased risk (10.96% and 15.53%, respectively). In the fully adjusted model, advanced CKM stages remained independent predictors of MINS, with stages 3 (OR: 1.95; 95% CI: 1.60-2.37) and 4 (OR: 2.16; 95% CI: 1.75-2.66) conferring significantly elevated risk. These findings challenge the conventional linear risk assumptions and highlight the unique risk patterns that emerge in the perioperative setting.

**Central Illustration:**
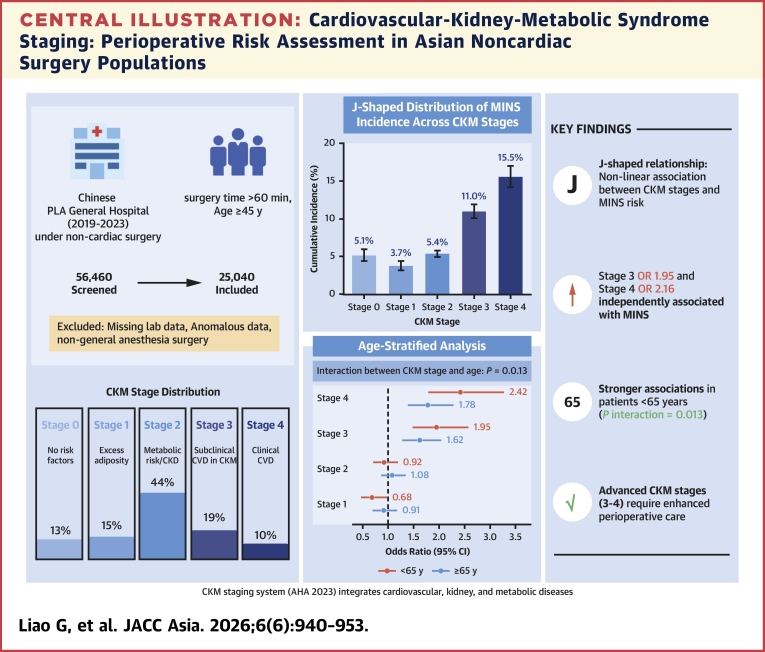
Cardiovascular-Kidney-Metabolic Syndrome Staging: Perioperative Risk Assessment in Asian Noncardiac Surgery Populations Overview of the association between CKM syndrome staging and MINS in 25,040 noncardiac surgical patients. A J-shaped relationship emerged: stage 1 showed the lowest MINS incidence (3.76%), whereas stages 3 and 4 demonstrated significantly elevated risk (adjusted OR: 1.95 and 2.16, respectively). Age-stratified analysis revealed stronger associations in younger patients (<65 years; *P* for interaction = 0.013). Stage 3 represents a critical threshold where metabolic dysfunction transitions to subclinical CVD, marking substantial perioperative risk escalation. CKM = cardiovascular-kidney-metabolic; CVD = cardiovascular disease; MINS = myocardial injury after noncardiac surgery.

The CKM stage distribution in the surgical cohort (stage 0: 13.0%, stage 1: 15.0%, stage 2: 43.7%, stage 3: 18.7%, and stage 4: 9.7%) demonstrated patterns generally consistent with contemporary population studies. Recent U.S. National Health and Nutrition Examination Survey data (2011-2020) showed stage distributions of 10.6%, 25.9%, 49.0%, 5.4%, and 9.2% for stages 0 to 4, respectively.^11^ The 2025 Chinese National Survey found stage distributions of 18.8%, 15.5%, 42.1%, 14.7%, and 8.9% for stages 0 to 4, with distributions similar to our surgical cohort.^12^ A systematic review of CKM prevalence across the Americas, Europe, and Western Pacific regions found comparable patterns, although surgery-specific data remained absent.^13^ The predominance of stage 2 patients across all cohorts reflects the widespread burden of metabolic dysfunction characterized by established risk factors including hypertension, diabetes, and kidney disease, representing a substantial population in suboptimal metabolic health.

The observed J-shaped CKM-MINS relationship is supported by recent perioperative research. Roth et al^6^ analyzed 14,634 European patients undergoing noncardiac surgery and reported similar patterns: major adverse cardiovascular events remained low across stages 0 to 2 (0.8%-1.4%) before increasing substantially in stages 3 and 4 (2.8%-4.7%). This J-shaped pattern contrasts with general population studies showing linear associations,^12^^,^^14^^,^^15^ suggesting that perioperative stress changes cardiovascular risk relationships. The transition point at stage 3, representing the progression from metabolic dysfunction to subclinical CVD, emerges as a critical threshold where the perioperative risk escalates dramatically.

The age-stratified analysis revealed particularly strong CKM-MINS associations in younger patients (<65 years), with ORs of 5.03 and 5.95 for stages 3 and 4, respectively, compared with 3.99 and 2.81 in older patients. This finding aligns with the emerging evidence that CKM components accelerate cardiovascular aging, with affected individuals developing an elevated risk 8 to 28 years earlier than those without CKM factors.^16^ Our findings are consistent with the results from Li et al^14^ in the Kailuan cohort study, which demonstrated that the association between CKM staging and MINS was stronger in younger patients (<65 years) than in older patients (≥65 years) (*P* for interaction <0.001). Stronger associations in younger patients likely reflect reduced competing risks and more direct CKM-mediated effects. Sex did not modify the CKM-MINS relationship, suggesting universal applicability across sexes. Among the surgical types, neurosurgery showed the strongest associations (stage 3: OR: 5.5), possibly because of prolonged operative times, prone positioning, and complex hemodynamic management requirements that particularly challenge patients with compromised cardiovascular reserves.^17^^,^^18^ Notably, several subgroups demonstrated the counterintuitive pattern in which stage 3 risk exceeded stage 4 risk, including patients ≥65 years (OR: 3.99 vs 2.82), urological surgery patients (OR: 4.91 vs 2.92), and hepatobiliary surgery patients (OR: 3.15 vs 3.08). This pattern may reflect several mechanisms. Stage 4 patients with established CVD receive enhanced perioperative cardiovascular monitoring and medical optimization, including intensive hemodynamic monitoring and evidence-based cardioprotective therapies.^19^^,^^20^ In addition, patients with known CVD undergoing elective surgery represent a preselected population that is hemodynamically stable and has undergone comprehensive cardiac evaluation.^18^ In contrast, stage 3 patients with subclinical CVD may have unrecognized vulnerability during perioperative stress, as they typically do not receive the intensive optimization and monitoring protocols provided to patients with established CVD.^3^^,^^21^

The protective effect observed in patients in stage 1 exemplifies the "obesity paradox" in surgical populations.^22^^,^^23^ This paradox occurs through several mechanisms. The adipose tissue provides metabolic reserves during surgical stress.^24^ Chronic inflammation may "precondition" immune responses to trauma.^25^ Moreover, adipokines exert cardioprotective effects.^26^ This protection occurs mainly in "metabolically healthy" obese patients who maintain insulin sensitivity.^27^ By definition, patients in stage 1 fit this protective profile.

However, when surgical variables were adjusted for the fully adjusted model, this protection disappeared. This suggests that perioperative factors attenuate the protective effects of the obesity paradox.^28^ By contrast, surgical stress poses a greater challenge in patients with advanced CKM stages. These patients experience harmful neuroendocrine responses, plaque destabilization, and hemodynamic stress that can overwhelm their cardiovascular reserve,^29^, ^30^, ^31^ resulting in the loss of stage 1 protective effects.

A shortcoming of the current CKM staging system is its classification of all individuals with BMI <23 kg/m^2^ and no metabolic risk factors into a single stage 0 "no risk" category. However, this grouping combines 2 subpopulations with fundamentally different risk profiles: truly healthy normal-weight individuals and underweight patients. Our sensitivity analysis confirmed that this classification masks important risk heterogeneity. Underweight patients demonstrated significantly higher MINS risk than both normal-weight stage 0 patients (OR: 1.72; *P* = 0.014) and stage 1 patients (OR: 2.20; *P* < 0.001), explaining the seemingly paradoxical finding that stage 0 exceeded stage 1 in overall MINS incidence. These patients often present with inadequate nutritional reserves, reduced muscle mass, and impaired immune function, leaving them vulnerable to surgical stress due to compromised physiological reserves.^32^, ^33^, ^34^

Beyond the established principle that advanced CKM stages (stage 3 and 4) require intensive perioperative cardiac management, our findings highlight 2 unique clinical considerations. First, the heterogeneity within stage 0 reveals that underweight patients (BMI <18.5 kg/m^2^), despite being classified as "no CKM risk," exhibited elevated MINS risk. This suggests that preoperative nutritional optimization and careful assessment of physiological reserve should be prioritized in this vulnerable subgroup, rather than assuming low risk based solely on CKM stage 0 classification. Second, the significant age interaction (*P* = 0.013) demonstrated that younger patients (<65 years) with advanced CKM stages showed substantially stronger risk associations compared with older patients. This finding suggests that younger surgical patients with CKM stage 3-4 may harbor unrecognized cardiovascular vulnerability and warrant heightened perioperative vigilance and care despite their age.

### Study limitations

The retrospective, single-center design may limit the generalizability of our results. Substantial exclusions owing to missing data (n = 30,122) introduced a potential selection bias. The absence of waist circumference measurements may have resulted in stage 1 underclassification. Reliance on routine clinical data rather than specialized cardiac imaging or biomarker assessments may have affected the precision of subclinical CVD detection, particularly for stage 3 CKM classification. Furthermore, the sensitivity analysis of underweight patients within stage 0 showed a trend toward increased MINS risk (OR: 1.49; *P* = 0.10) that did not reach statistical significance, potentially due to limited sample size (n = 341). Further investigation in larger cohorts is warranted to validate this finding. However, despite these limitations, this study suggests the utility of CKM staging in perioperative risk assessment and identifies stage 3 as a critical risk threshold for surgical populations.

## Conclusions

The CKM staging system demonstrated significant independent associations with MINS risk, exhibiting a distinctive J-shaped relationship. Advanced CKM stages are associated with an increased risk of MINS, with the most dramatic escalation occurring during the transition from metabolic dysfunction to subclinical CVD. This association was particularly clear in younger patients and remained consistent across diverse surgical specialties. These findings may improve perioperative risk assessment and guide preventive strategies. Future optimization of CKM staging for surgical populations should consider distinguishing between underweight and normal-weight patients in stage 0.

### Data Availability

The datasets used and/or analyzed during the current study are not publicly available because of institutional data protection policies but are available from the corresponding author on reasonable request.

## Funding Support and Author Disclosures

This research was supported by the National Key Research and Development Program of China (2018YFC2001900). The authors have reported that they have no relationships relevant to the contents of this paper to disclose.
