# Supplementary material for: Association Between Cardiovascular-Kidney-Metabolic Syndrome and Myocardial Injury After Noncardiac Surgery: A Retrospective Cohort Study
Source: JACC Asia. 2026 Mar 14;6(6):940–53. doi: 10.1016/j.jacasi.2026.01.023 (PMC13244111; doi:10.1016/j.jacasi.2026.01.023)
Supplement: Supplemental Figures 1 and 2 and Supplemental Tables 1-7 [file mmc1.docx]

**Supplemental Table 1. Univariate Analysis of Baseline Risk Factors for MINS**

| **Variables** | **MINS Group (n=1,782)** | **No MINS Group (n=23,258)** | **OR** | **95% CI** | **P Value** |
| --- | --- | --- | --- | --- | --- |
| ***CKM Stage*** | | | | | |
| CKM Stage 1 (vs Stage 0) | 141 (3.8%) | 3,613 (96.2%) | 0.724 | 0.576–0.911 | **0.006** |
| CKM Stage 2 (vs Stage 0) | 586 (5.4%) | 10,357 (94.6%) | 1.050 | 0.880–1.253 | 0.590 |
| CKM Stage 3 (vs Stage 0) | 512 (11.0%) | 4,158 (89.0%) | 2.285 | 1.906–2.738 | **<0.001** |
| CKM Stage 4 (vs Stage 0) | 377 (15.5%) | 2,050 (84.5%) | 3.412 | 2.819–4.130 | **<0.001** |
| ***Demographics*** | | | | | |
| Age (years) | 67.54 ± 10.03 | 61.25 ± 8.97 | 1.075 | 1.069–1.081 | **<0.001** |
| Height (cm) | 166.54 ± 7.87 | 166.30 ± 7.91 | 1.004 | 0.998–1.010 | 0.206 |
| Weight (kg) | 67.22 ± 12.08 | 68.77 ± 11.69 | 0.989 | 0.984–0.993 | **<0.001** |
| BMI (kg/m²) | 24.17 ± 3.62 | 24.80 ± 3.39 | 0.946 | 0.932–0.960 | **<0.001** |
| Gender (Male) | 557 (5.4%) | 9,791 (94.6%) | 0.625 | 0.564–0.694 | **<0.001** |
| Smoking history | 607 (8.8%) | 6,320 (91.2%) | 1.385 | 1.250–1.533 | **<0.001** |
| Alcohol history | 559 (7.2%) | 7,233 (92.8%) | 1.013 | 0.913–1.123 | 0.813 |
| ***Comorbidities*** | | | | | |
| Hypertension | 900 (9.2%) | 8,874 (90.8%) | 1.654 | 1.502–1.822 | **<0.001** |
| Diabetes mellitus | 460 (10.2%) | 4,065 (89.8%) | 1.643 | 1.470–1.836 | **<0.001** |
| Hyperlipidemia | 6 (3.5%) | 166 (96.5%) | 0.470 | 0.208–1.063 | 0.070 |
| Hyperlipidemia | 30 (7.5%) | 372 (92.5%) | 1.053 | 0.724–1.533 | 0.786 |
| Cardiovascular disease | 377 (15.5%) | 2,050 (84.5%) | 2.776 | 2.456–3.137 | **<0.001** |
| ***Laboratory Parameters*** | | | | | |
| Serum creatinine (mg/dL) | 1.10 ± 1.02 | 0.84 ± 0.26 | 2.758 | 2.434–3.125 | **<0.001** |
| Serum creatinine (μmol/L) | 96.82 ± 90.10 | 74.54 ± 23.16 | 1.012 | 1.010–1.013 | **<0.001** |
| Preoperative fasting glucose (mmol/L) | 5.93 ± 2.11 | 5.46 ± 1.45 | 1.162 | 1.134–1.190 | **<0.001** |
| Preoperative total cholesterol (mmol/L) | 4.11 ± 1.06 | 4.39 ± 0.96 | 0.734 | 0.697–0.774 | **<0.001** |
| Preoperative triglycerides (mmol/L) | 1.48 ± 0.86 | 1.52 ± 0.89 | 0.940 | 0.887–0.996 | **0.037** |
| Neutrophil count (×10⁹/L) | 0.64 ± 0.12 | 0.58 ± 0.10 | 185.979 | 117.159–295.223 | **<0.001** |
| Platelet count (×10⁹/L) | 220.04 ± 76.76 | 223.34 ± 67.85 | 0.999 | 0.999–1.000 | 0.050 |
| ***Surgical Characteristics*** | | | | | |
| Surgery duration (minutes) | 257.94 ± 143.42 | 194.82 ± 105.54 | 1.004 | 1.004–1.004 | **<0.001** |
| Urine output (mL) | 680.74 ± 748.13 | 562.39 ± 637.74 | 1.000 | 1.000–1.000 | **<0.001** |
| Blood loss (mL) | 393.51 ± 858.30 | 152.57 ± 263.10 | 1.001 | 1.001–1.001 | **<0.001** |
| Intraoperative hypotension | 1,170 (8.1%) | 13,309 (91.9%) | 1.429 | 1.292–1.581 | **<0.001** |
| ASA Class 2 (vs Class 1) | 1,126 (5.3%) | 20,179 (94.7%) | 0.262 | 0.236–0.290 | **<0.001** |
| ASA Class 3 (vs Class 1) | 590 (18.6%) | 2,579 (81.4%) | 3.969 | 3.567–4.416 | **<0.001** |
| ASA Class 4 (vs Class 1) | 59 (49.6%) | 60 (50.4%) | 13.239 | 9.212–19.027 | **<0.001** |

This analysis includes only variables present in the baseline characteristics table. Continuous variables are presented as mean ± standard deviation. Categorical variables are presented as number (percentage). P < 0.05 was considered statistically significant.Red text indicates statistically significant results (P < 0.05). Blue background indicates CKM stage comparisons.

**Supplemental Table 2. Sensitivity Analysis of CKM Stage 0 Subgroups**

*Comparison of MINS Risk Among Underweight vs. Normal Weight Patients Within CKM Stage 0*

***A. MINS Incidence Rates by Group***

| **Group** | **Sample Size** | **MINS Events** | **MINS Rate (%)}}** | **95% CI** |
| --- | --- | --- | --- | --- |
| **CKM Stage 0 Subgroups by BMI** | | | | |
| Normal Weight (Reference) | 2,905 | 139 | 4.78 | 4.07–5.62 |
| Underweight (BMI <18.5 kg/m²) | 341 | 27 | 7.92 | 5.50–11.27 |
| **Comparison Group** | | | | |
| CKM Stage 1 | 3,754 | 141 | 3.76 | 3.19–4.41 |

***B. Multivariable Logistic Regression Analysis (Four Progressive Models)***

| **Comparison** | **Model 1 (Unadjusted)** | | | **Model 2 (Preoperative)** | | | **Model 3 (Surgery)** | | | **Model 4 (Fully Adjusted)** | | |
| --- | --- | --- | --- | --- | --- | --- | --- | --- | --- | --- | --- | --- |
|  | **OR** | **95% CI** | **P** | **OR** | **95% CI** | **P** | **OR** | **95% CI** | **P** | **OR** | **95% CI** | **P** |
| Normal (Ref) | *1.00* | *—* | *—* | *1.00* | *—* | *—* | *1.00* | *—* | *—* | *1.00* | *—* | *—* |
| Underweight | 1.72 | 1.12–2.63 | **0.01** | 1.53 | 0.99–2.38 | 0.06 | 1.52 | 0.95–2.41 | 0.08 | 1.49 | 0.93–2.38 | 0.10 |
| CKM Stage 1 | 0.78 | 0.61–0.99 | **0.04** | 0.83 | 0.65–1.05 | 0.12 | 0.88 | 0.68–1.13 | 0.32 | 0.93 | 0.72–1.21 | 0.60 |

***C. Pairwise Statistical Comparisons (Unadjusted)***

| **Comparison** | **Statistical Test** | **OR** | **95% CI** | **P Value** |
| --- | --- | --- | --- | --- |
| Underweight vs. Normal Weight | Chi-square | 1.71 | 1.12–2.63 | **0.014** |
| Underweight vs. Stage 1 | Fisher's exact | 2.20 | 1.44–3.38 | **<0.001** |
| Normal Weight vs. Stage 1 | Fisher's exact | 1.29 | 1.01–1.64 | **0.042** |

***D. BMI Distribution Within CKM Stage 0 (n=3,246)***

| **BMI Category** | **Definition** | **Sample Size** | **Percentage** | **MINS Rate (%)** |
| --- | --- | --- | --- | --- |
| Underweight | <18.5 kg/m² | 341 | 10.5% | 7.92 |
| Normal Weight | 18.5–22.9 kg/m² | 2,905 | 89.5% | 4.78 |
| Overweight/Obese* | ≥23.0 kg/m² | 0 | 0.0% | — |

**Model Specifications:** Model 1 was unadjusted. Model 2 was adjusted for age, sex, lymphocyte count, neutrophil count, and platelet count. Model 3 was adjusted for operation duration, blood loss, ASA classification, hypotension events, and surgery type. Model 4 was fully adjusted for all covariates in Models 2 and 3.

**BMI Categories:** Asian-specific BMI cutoffs were used: underweight (<18.5 kg/m²), normal weight (18.5–22.9 kg/m²). *By definition, patients with BMI ≥23.0 kg/m² are classified as at least CKM Stage 1.

**Statistical Methods:** Logistic regression was performed with normal-weight Stage 0 patients as the reference group. Chi-square test and Fisher's exact test were used for pairwise comparisons. 95% confidence intervals for incidence rates were calculated using Wilson score method.

**Abbreviations:** MINS = myocardial injury after non-cardiac surgery; CKM = cardiovascular-kidney-metabolic; OR = odds ratio; CI = confidence interval; BMI = body mass index; ASA = American Society of Anesthesiologists; Ref = reference.

**Color Coding:** Yellow = underweight subgroup; Blue = normal-weight reference; Gray = comparison groups. **Red text** indicates P < 0.05.

This analysis examines CKM Stage 0 heterogeneity by BMI subgroups. P < 0.05 was considered statistically significant. Red text indicates statistically significant results (P < 0.05). Background colors indicate different BMI subgroups within Stage 0.
